# Supplementary figures and images for: Presence of Triatoma breyeri (Reduviidae, Triatominae) in Bolivia
Source: PLoS One. 2024 Jul 26;19(7):e0307989. doi: 10.1371/journal.pone.0307989 (PMC11280137; doi:10.1371/journal.pone.0307989)

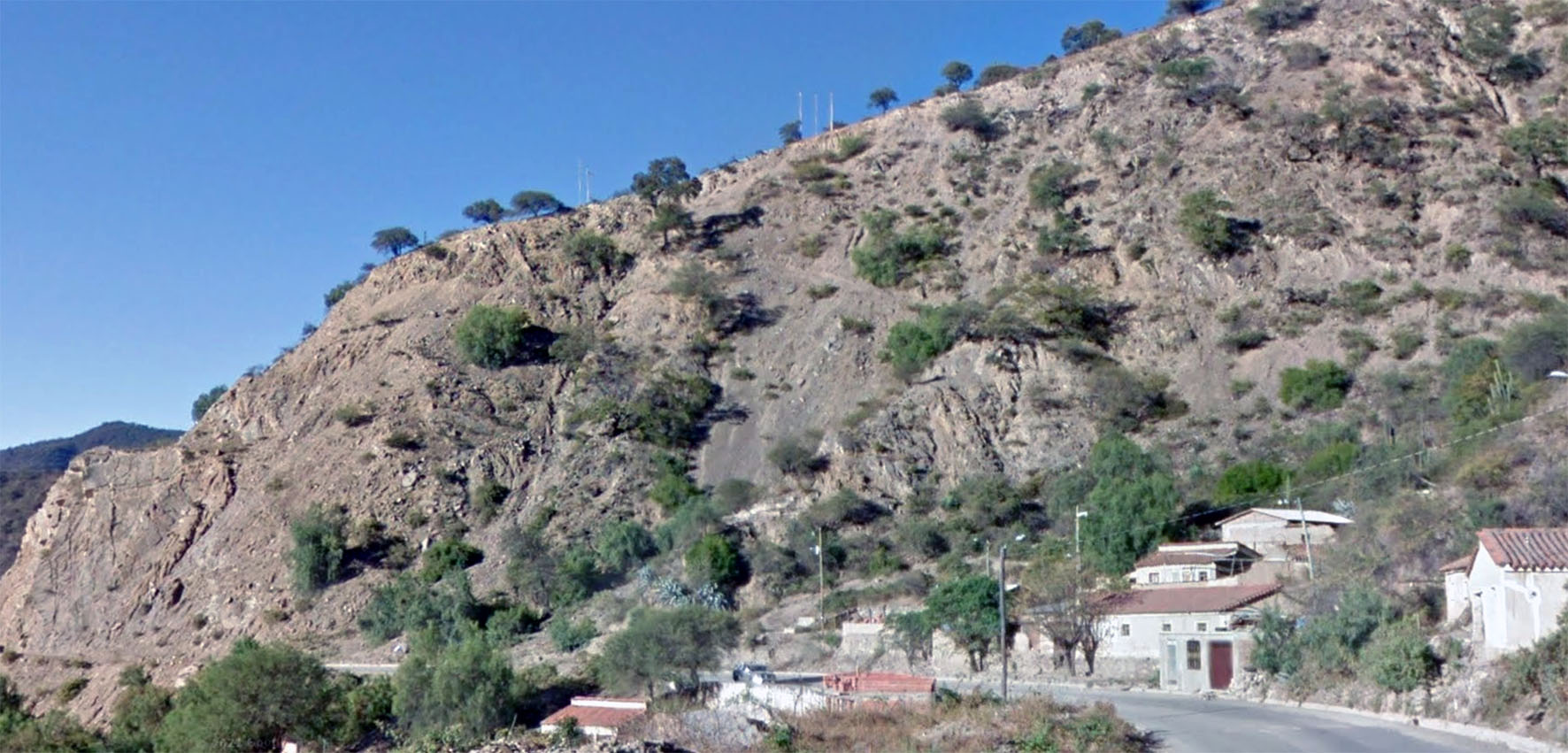

Supplement: S1 Fig — (JPG) [file pone.0307989.s001.jpg]

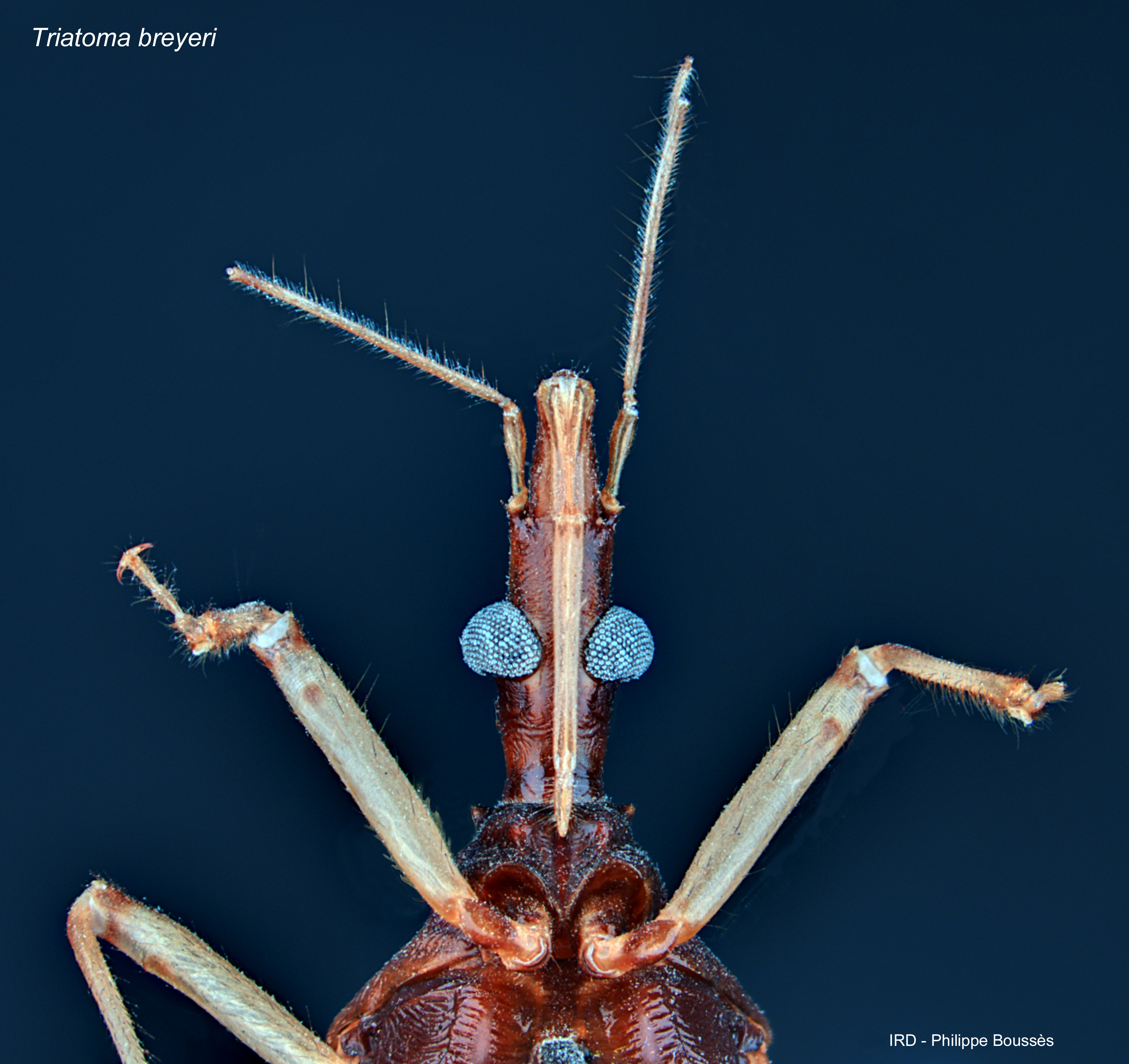

Supplement: S2 Fig — The specimen depicted is the one used for molecular identification and is named isolate A in the present study. (JPG) [file pone.0307989.s002.jpg]

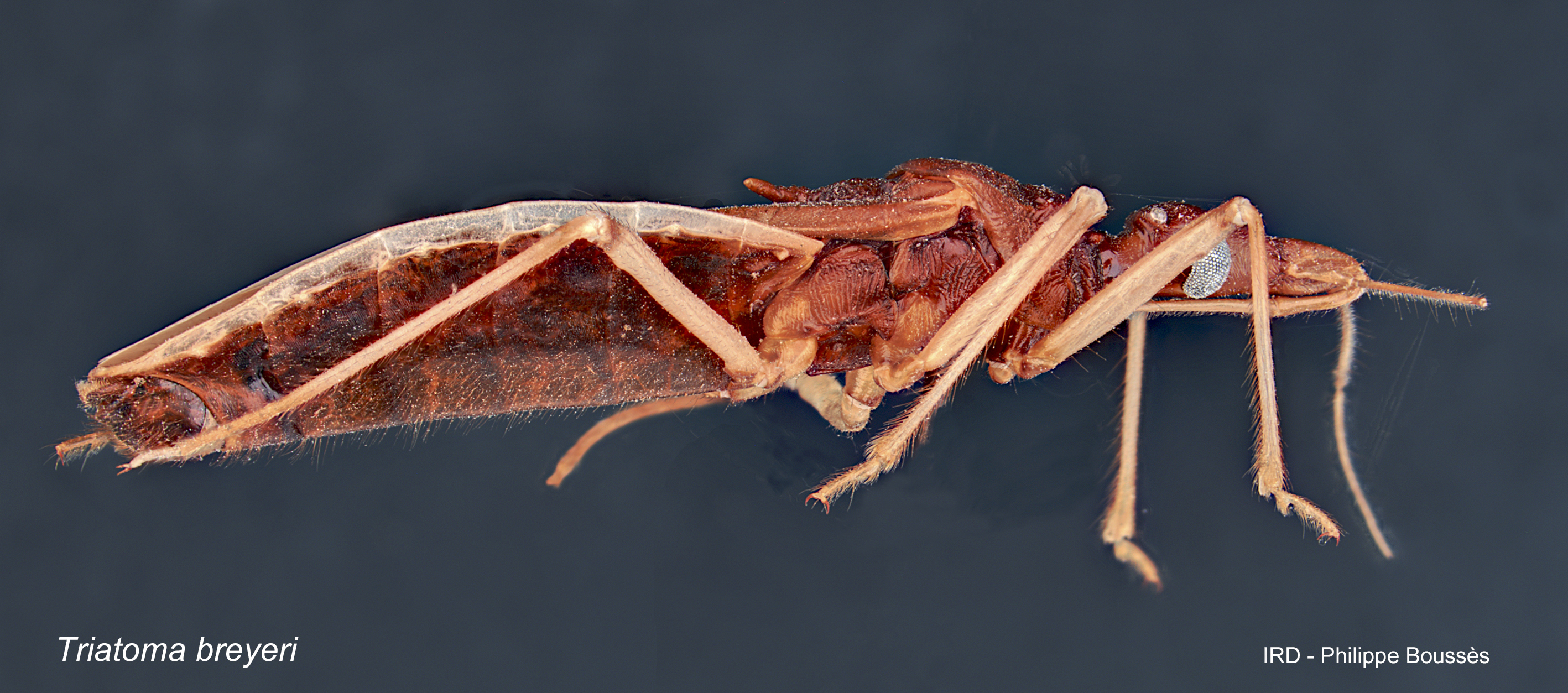

Supplement: S3 Fig — The specimen depicted is the one used for molecular identification and is named isolate A in the present study. (TIF) [file pone.0307989.s003.tif]

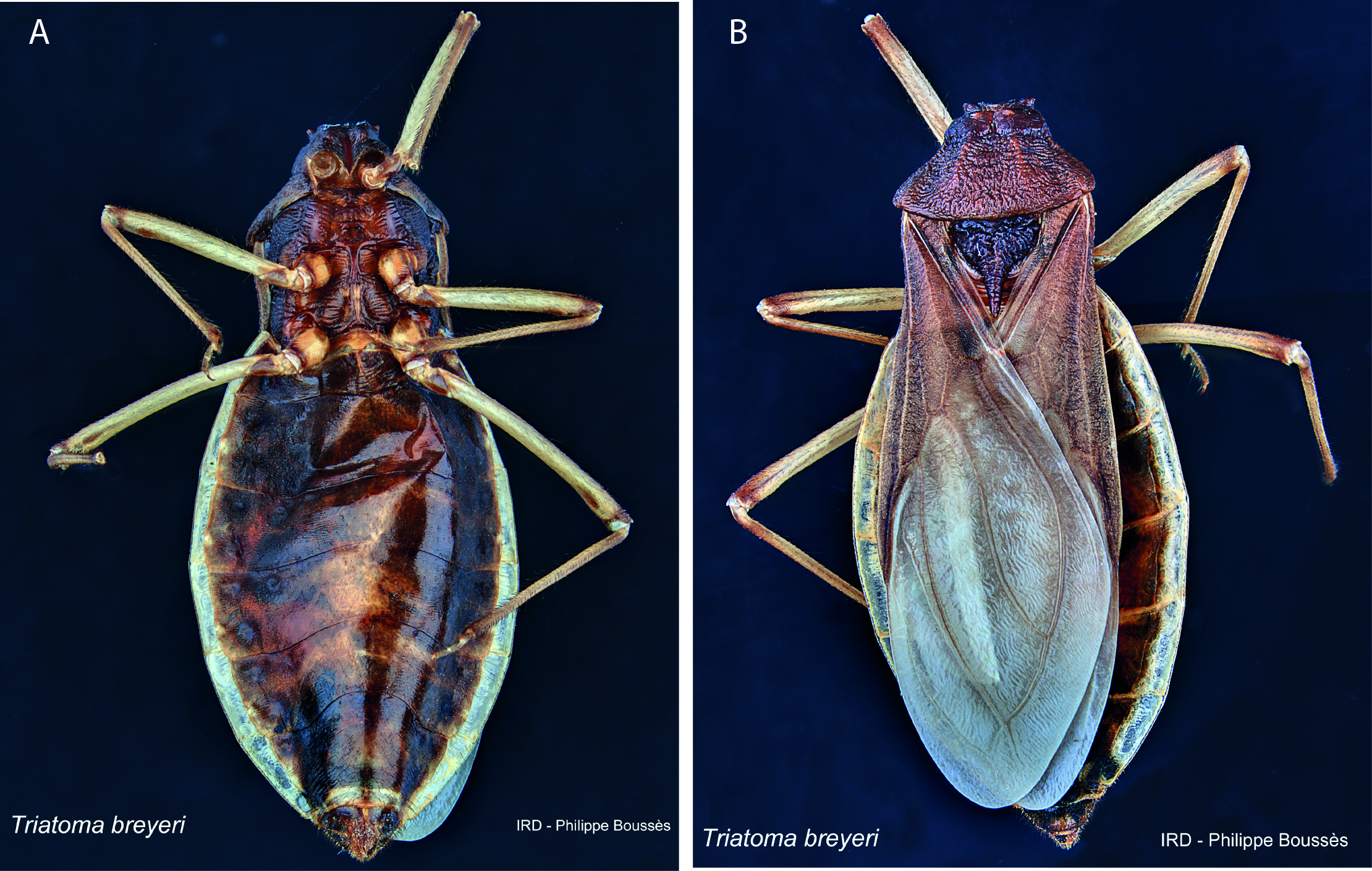

Supplement: S4 Fig — Ventral view (A) and dorsal view (B) of a specimen of Triatoma breyeri from Chuqui-Chuqui, Bolivia. The specimen depicted is the one used for molecular identification and is named isolate B in the present study. Head is missing. (TIF) [file pone.0307989.s004.tif]

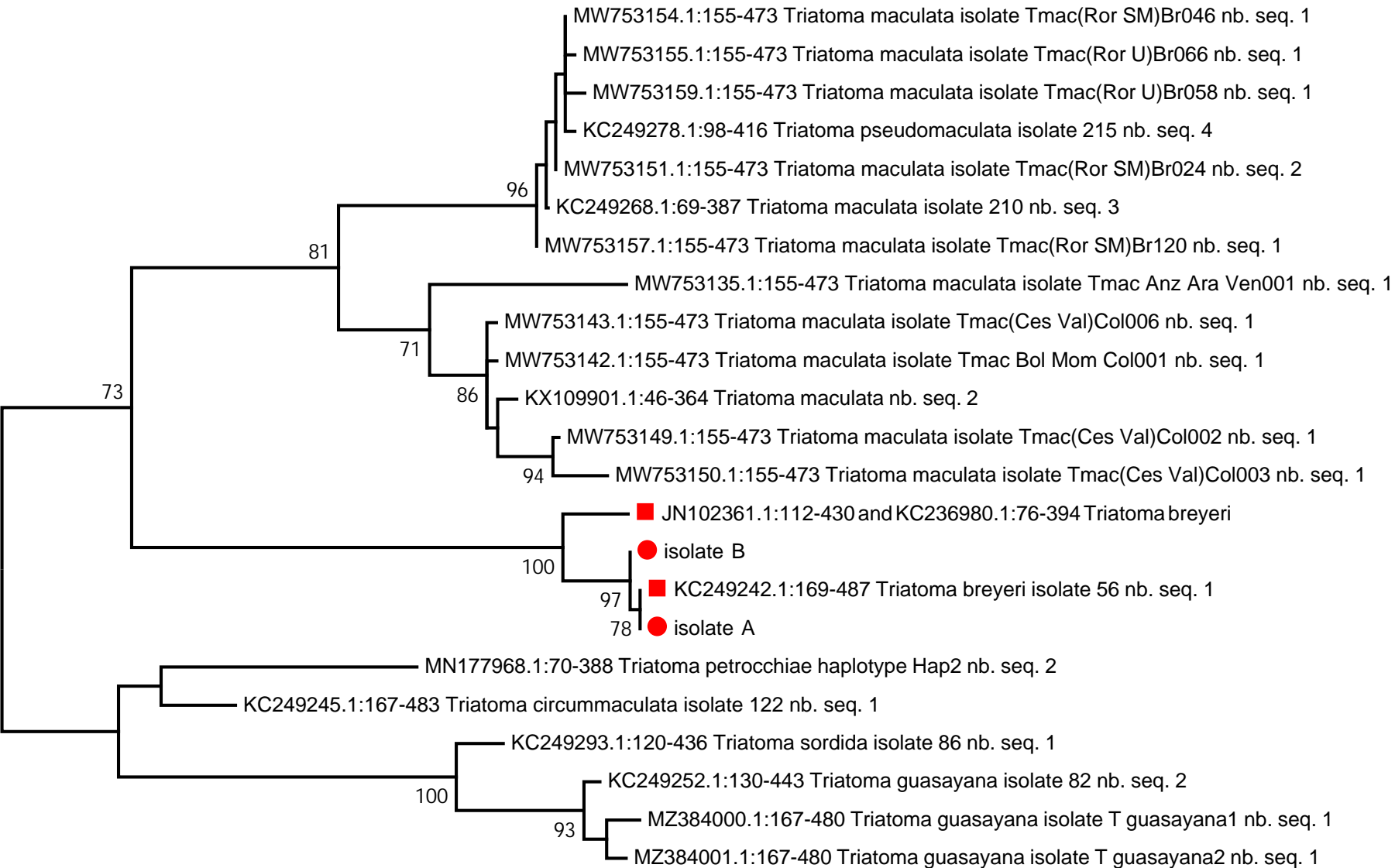

0.05

Supplement: S5 Fig — The numbers at the nodes correspond to the bootstrap values > 70%. The best multiple substitution model was the Tamura-Nei model +G (Gamma distribution) + I (invariants). Isolates A and B are visualized as red circles while T. breyeri reference sequences are represented as red squares. “nb. seq.” is the amount of the particular haplotype in GenBank. (PDF) [file pone.0307989.s005.pdf]

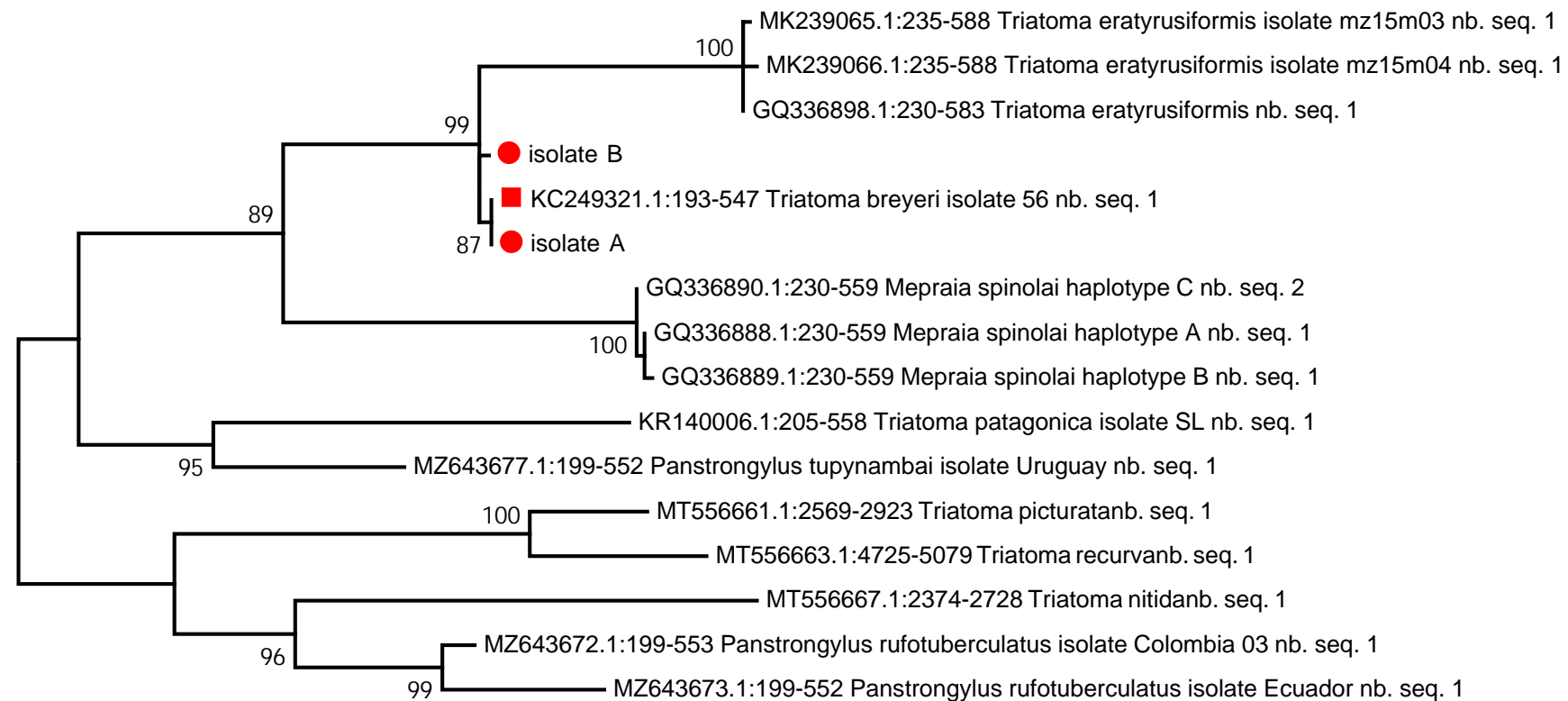

0.05

Supplement: S6 Fig — The numbers at the nodes correspond to the bootstrap values > 70%. The best multiple substitution model was the GTR model +G (Gamma distribution) + I (invariants). Isolates A and B are visualized as red circles while the only T. breyeri reference sequence is represented as a red square. “nb. seq.” is the amount of the particular haplotype in GenBank. (PDF) [file pone.0307989.s006.pdf]

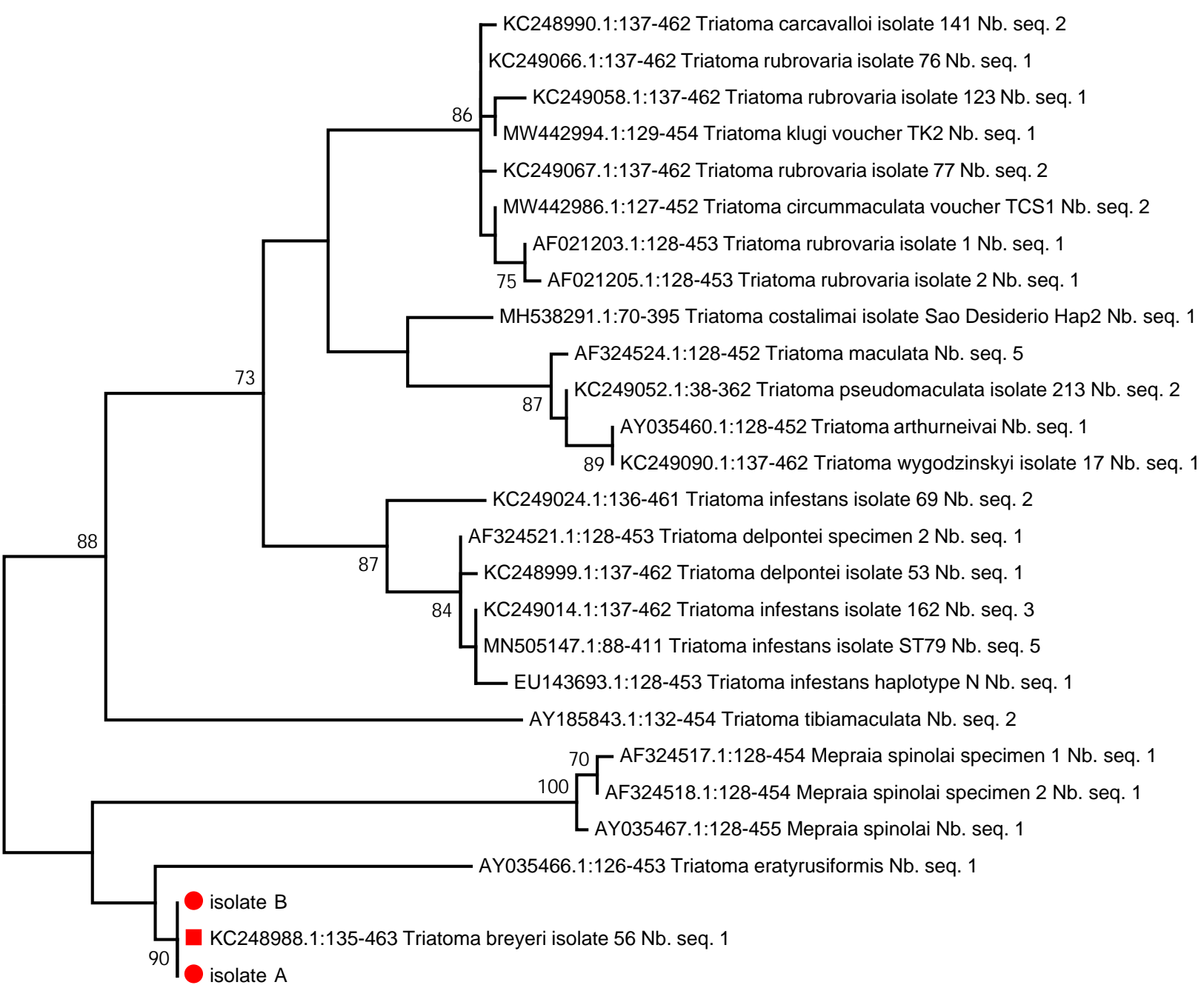

0.02

Supplement: S7 Fig — The numbers at the nodes correspond to the bootstrap values > 70%. The best multiple substitution model used was the Hasegawa-Kishino-Yano mode (HKY) model +G (Gamma distribution). Isolates A and B are visualized as red circles while the only T. breyeri reference sequence is represented as a red square. “nb. seq.” is the amount of the particular haplotype in GenBank. (PDF) [file pone.0307989.s007.pdf]

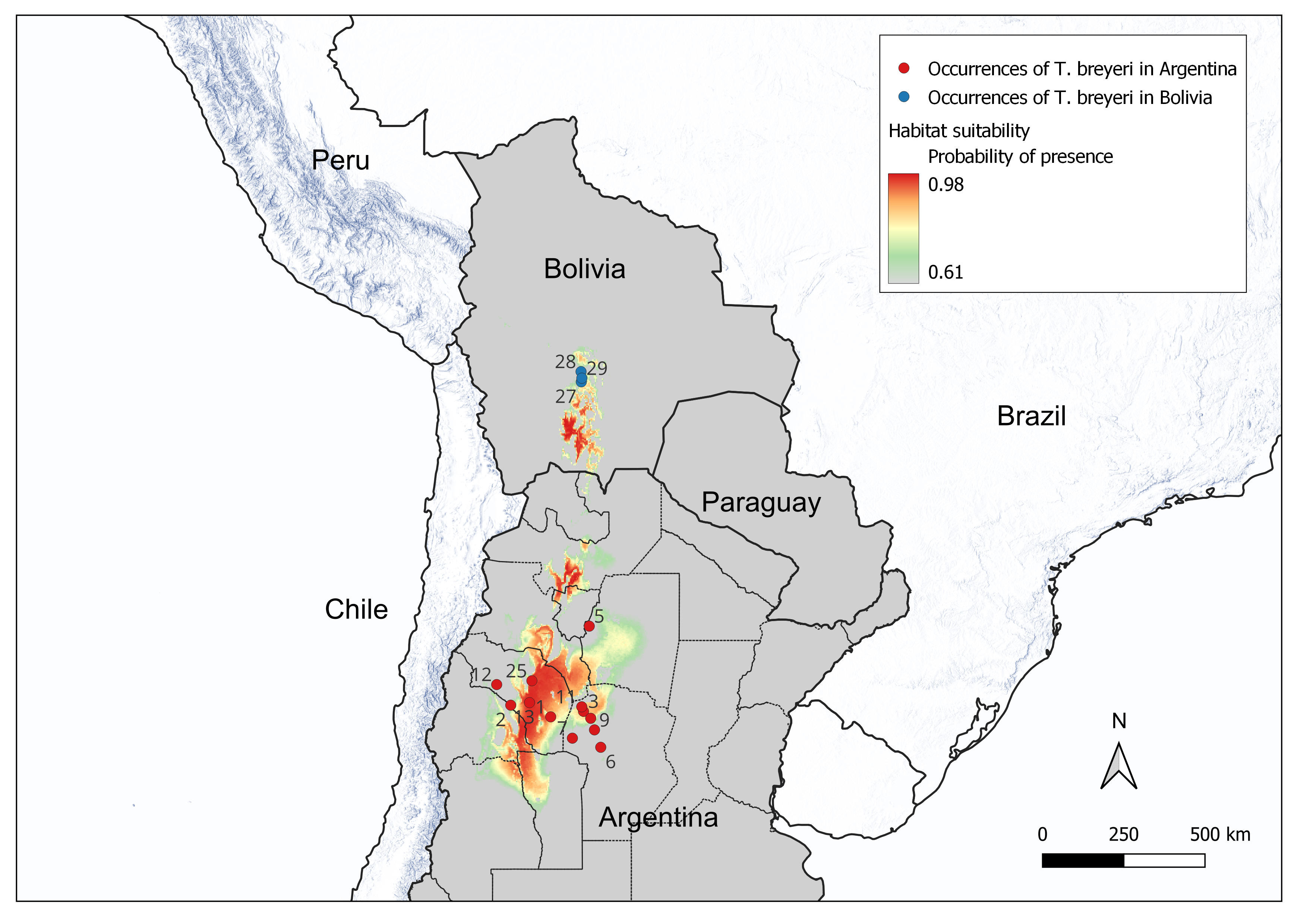

Supplement: S8 Fig — MaxEnt output is the Complementary Log-Log (cloglog) format which is a probability of presence. The mean maximum sensitivity plus specificity (MaxTSS) Cloglog threshold was used to depict in grey values below it (0.61 in the study). Numbers in the map refer to Id number of Table 1. Country contours were downloaded from the GADM website, and the data are classified as public. (TIF) [file pone.0307989.s008.tif]

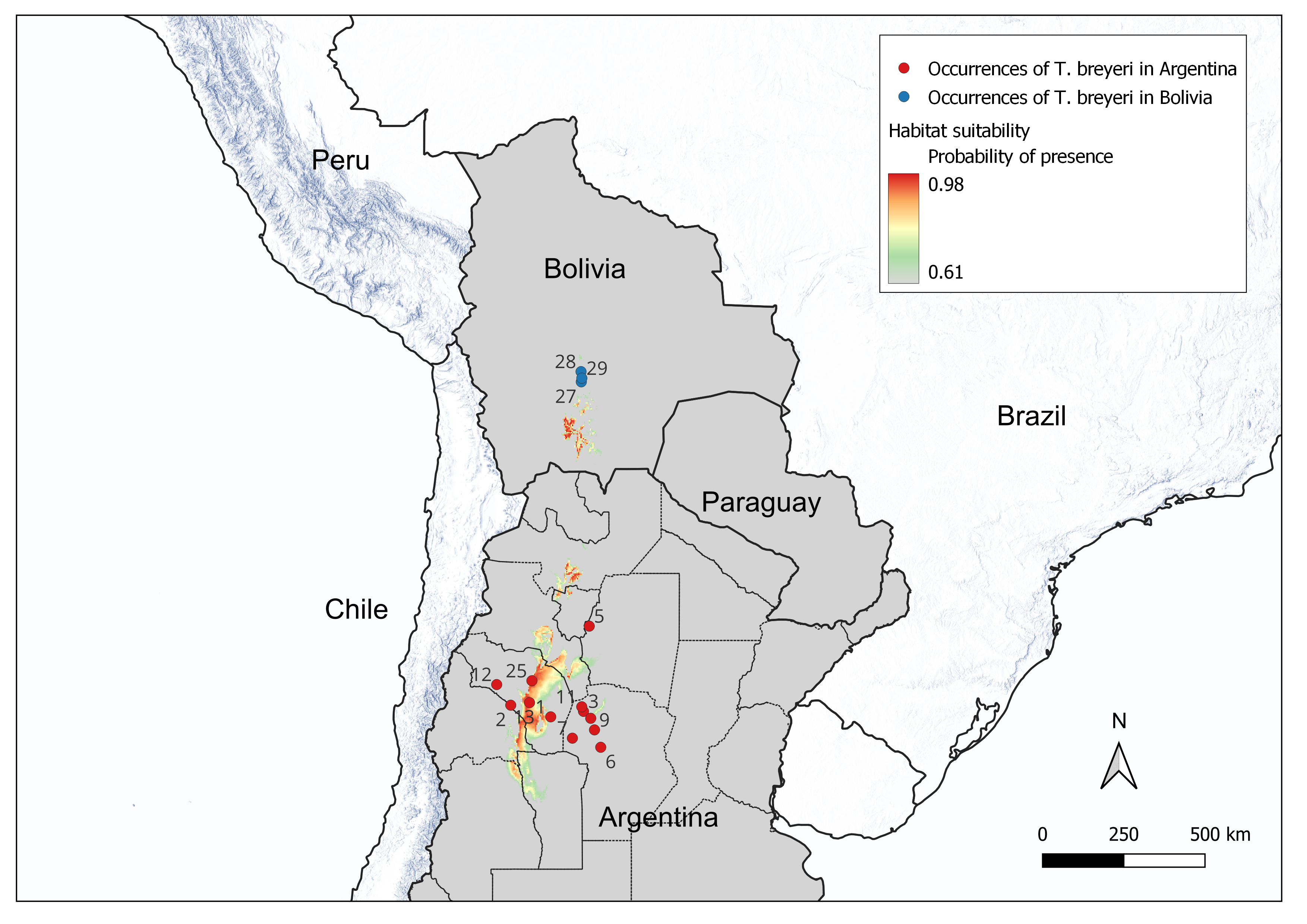

Supplement: S9 Fig — MaxEnt output is the Complementary Log-Log (cloglog) format which is a probability of presence. The mean maximum sensitivity plus specificity (MaxTSS) Cloglog threshold was used to depict in grey values below it (0.63 in the study). Numbers in the map refer to Id number of Table 1. Country contours were downloaded from the GADM website, and the data are classified as public. (TIF) [file pone.0307989.s009.tif]

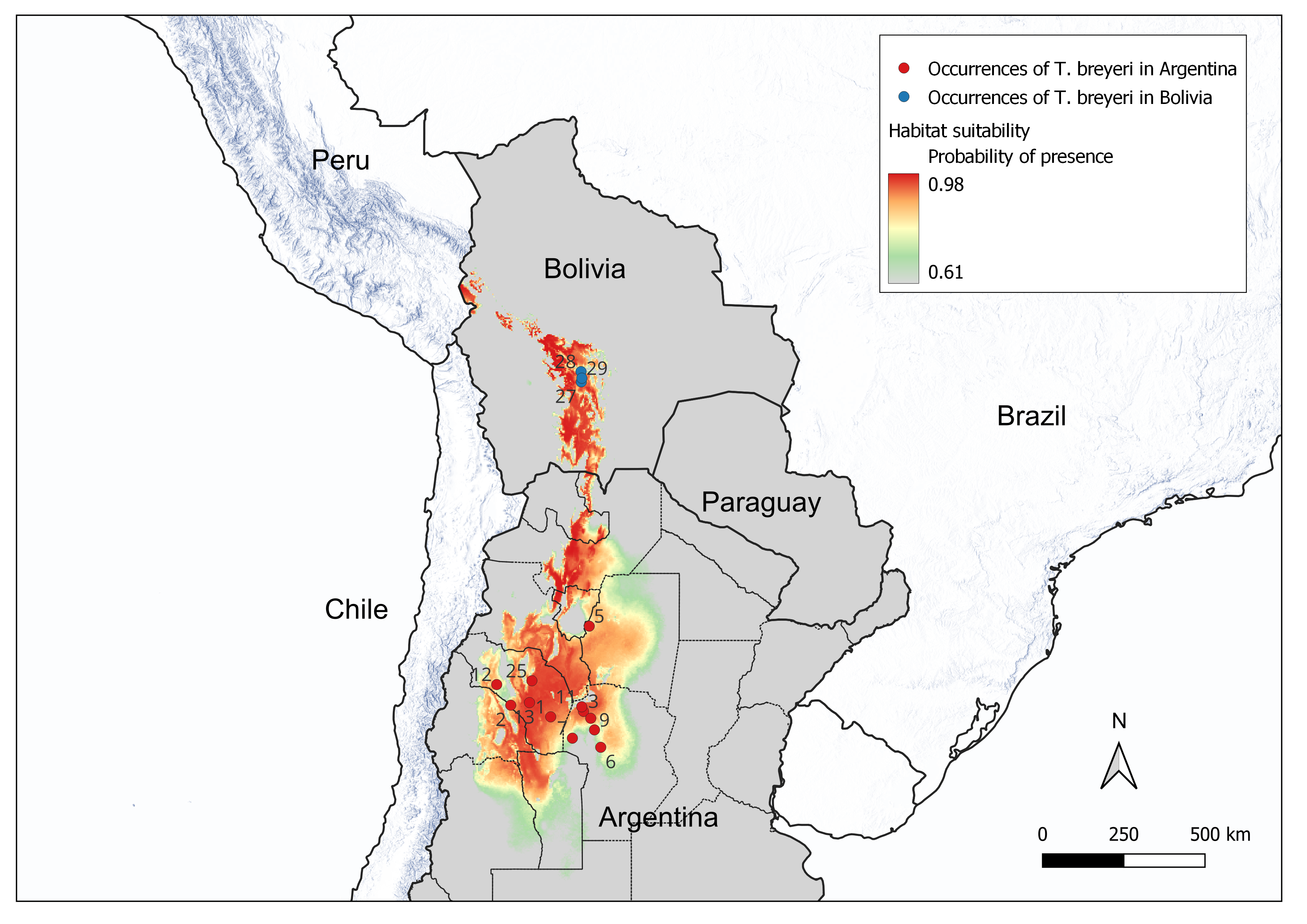

Supplement: S10 Fig — MaxEnt output is the Complementary Log-Log (cloglog) format which is a probability of presence. The mean maximum sensitivity plus specificity (MaxTSS) Cloglog threshold was used to depict in grey values below it (0.63 in the study). Numbers in the map refer to Id number of Table 1. Country contours were downloaded from the GADM website, and the data are classified as public. (TIF) [file pone.0307989.s010.tif]
