# Supplementary material for: Presence of Triatoma breyeri (Reduviidae, Triatominae) in Bolivia
Source: PLoS One. 2024 Jul 26;19(7):e0307989. doi: 10.1371/journal.pone.0307989 (PMC11280137; doi:10.1371/journal.pone.0307989)
Supplement: S2 Table — VIF values are from the step procedure carried out with the vifstep function of the R-package sdm, with threshold = 5. Correlations are values at the end of the vifcor procedure carried out with the sdm R-package with threshold = 0.7. (DOCX) [file pone.0307989.s012.docx]

|  |  |  | **Correlations** | | | |
| --- | --- | --- | --- | --- | --- | --- |
| **Variable** | **Definition** | **VIF** | **BIO2** | **BIO4** | **BIO6** | **BIO13** |
| **BIO2** | Mean Diurnal Range  (Mean of monthly (max temp - min temp)) | 3.16 |  |  |  |  |
| **BIO4** | Temperature Seasonality  (Standard deviation ×100) | 3.10 | 0.40 |  |  |  |
| **BIO6** | Min Temperature of Coldest Month | 3.45 | -0.58 | 0.27 |  |  |
| **BIO13** | Precipitation of Wettest Month | 1.91 | -0.60 | -0.35 | 0.45 |  |
| **BIO17** | Precipitation of Driest Quarter | 2.06 | -0.56 | -0.54 | 0.27 | 0.53 |
